# Syrupus Ferri Iodidi

**Published:** 1868

**Authors:** Edward R. Squibb


Syrupus Ferri Iodidi. By Edward R. Squibb, M. D.

The difficulty of keeping this preparation without change has again been discussed of late in the *London Pharmaceutical Journal*, and the insufficiency of the various plans resorted to have been pretty well shown in these discussions, and in the communications elicited thereby.

In a very considerable experience with the last official process, (U. S. P.,) the writer has only once seen the syrup become discolored. In this instance a syrup made in September and put up in pound bottles was found to be all somewhat discolored, though not uniformly so, and all shading off from the surface downward, in January following. This single instance, however, proves that even under ordinary good care, and nearly uniform management, some slight accident may determine a liberation of iodine. Dusty bottles, the use of corks instead of glass stoppers, and many other such apparently trivial matters, will often start or favor this change, though no such causes can be found in the instance referred to.

In estimating the amount of iodine liberated in this change, it was found to be exceedingly small, and practically quite insignificant, even in those bottles which were of the deepest color. No deposit had occurred in any of the bottles, however, and it is doubted whether a deposit ever occurs in any well managed preparation made by any of the later processes. It having been thus shown that the medicinal properties were not materially impaired by this change, since the amount of free iodine was too minute to produce any effect even upon the most delicate condition, a remedy for the discoloration was sought for upon chemical principles, and was soon found in the hyposulphite of soda, and it is the object of this note to publish a method of using the remedy. A solution of fifteen or twenty grains of crystalized hyposulphite of soda in one fluid-ounce of water is strong enough for the purpose, and from fifteen to twenty minims of this solution is sufficient for each pound of the syrup, when the latter is not of a darker color than brown sherry wine. When darker than this, double the quantity is required. The solution is simply added to the discolored syrup in the bottles and shaken up at ordinary temperatures. Syrup so restored is afterward much less susceptible to the change, and the writer has now a specimen which has been freely exposed to the air in a graduated measure for four weeks or more, without any appearance of change even on the surface. The quantity of the solution required to restore the syrup is a measure of the amount of change which may have taken place. The restored syrup is not of the full original green color, but rather more inclined to olive, or a very pale brown, and is not quite as transparent. This latter is probably due to the precipitation of a minute quantity of finely divided sulphur.

This remedy having succeeded in one single instance, is considered reliable only so far as its chemical principles are concerned, and is only published in order that its applicability may be tested; and the writer suggests to those interested in the subject, who have time to investigate it practically, to ascertain whether the addition of a minute proportion of hyposulphite of soda to freshly made syrup would not secure it against change.

*Brooklyn, Feb.* 1, 1868.

